# Cyclosporine-induced alopecia:a case report, FDA adverse event reporting system analysis and literature assessment

**DOI:** 10.3389/fphar.2024.1453034

**Published:** 2024-08-28

**Authors:** Ying Wang, Youhong Wang, Ping Xu

**Affiliations:** ^1^ Department of Pharmacy, the Second Xiangya Hospital of Central South University, Changsha, China; ^2^ Institute of Clinical Pharmacy, Central South University, Changsha, China; ^3^ Department of Pharmacy, Xiamen Children’s Hospital (Children’s Hospital of Fudan University at Xiamen), Xiamen, China; ^4^ Fujian Key Laboratory of Neonatal Diseases, Xiamen Key Laboratory of Neonatal Diseases, Xiamen Children’s Hospital (Children’s Hospital of Fudan University at Xiamen), Xiamen, China

**Keywords:** alopecia, drug adverse event, cyclosporine, immune, case report

## Abstract

Cyclosporine is a potent immunosuppressive drug for various immune-mediated diseases in children. Cyclosporine’s expected therapeutic effect also carries a wide range of side effects. One of the most common and intriguing dermatological side effects is hypertrichosis. However, recent reports have recognized alopecia as a potential adverse effect of cyclosporine. Here, we report a case of a 29-month-old boy diagnosed with aplastic anemia. During cyclosporine therapy, the patient presented with hair loss on the scalp, which and subsequently spread to the eyebrows and eyelashes. The alopecic symptoms were not relieved following topical minoxidil liniment interventions. When the cyclosporine was discontinued, a remarkable improvement was observed in the scalp, with complete hair regrowth. Data concerning cyclosporine from the FDA Adverse Event Reporting System (FAERS) database were extracted from January 2004 to January 2023. Within FAERS, our post-marketing pharmacovigilance analysis detected the reporting association of cyclosporine and alopecia. In monotherapy, cyclosporine-induced alopecia was observed in 118 cases, and tacrolimus-induced alopecia signals were detected in 197 cases. Although the potential mechanism of medication-induced hair loss is unclear, we identified a potential correlation between alopecia and cyclosporine, and it is still necessary to adequately recognize and clinically monitor this paradoxical reaction.

## Introduction

Aplastic anemia (AA) is a rare, heterogeneous, and potentially fatal disorder characterized by pancytopenia with hypocellular bone marrow. Radiation, chemical exposure, infection, and genetic factors are major etiologies of aplastic anemia (Gale et al., 2023). For children with aplastic anemia, anti-thymocyte globulin (ATG) combined with cyclosporine (CsA) is currently first-line therapy in the absence of a matched sibling donor hematopoietic stem cell transplantation (MRD-HSCT), with a long-term survival rate of approximately 87%–92% (Peinemann et al., 2013; Dufour et al., 2015; Samarasinghe et al., 2018).

CsA, an immunosuppressive calcineurin inhibitor, treats various immune-mediated diseases (Survase et al., 2011; Gui et al., 2021). The mechanism of action involves a combination with calcineurin. CsA prevents the transport of nuclear factor of activated T-cells by inhibiting calcineurin activity, thus suppressing the immune response by downregulating the transcription of interleukin-2, interferon-gamma and other cytokine genes (Survase et al., 2011). The expected therapeutic effect of CsA also carries a wide range of side effects, such as nephrotoxicity, hypertension, hepatotoxicity and neurotoxicity (Sternthal et al., 2008). CsA acts as a potent hair growth stimulator, more than 88% of people develop hypertrichosis following treatment (Panicker et al., 2012; Hawkshaw and Paus, 2021). This adverse effect has been considered an adjuvant therapy to enhance human hair growth. Recently, it has been demonstrated that CsA is effective in monotherapy or combination with systemic corticosteroids for alopecia areata. As a result of an excellent clinical response rate (57.02%–69.41%), CsA is used off-label in alopecia areata (Nowaczyk et al., 2020; Rudnicka et al., 2024). However, given the increase in its use, CsA-induced hair loss is suspected to be a new adverse drug reaction (Ezemma et al., 2024). Here, we present a rare case of a child who, during CsA therapy for severe aplastic anemia, suddenly developed severe hair loss. With medical intervention, the child’s alopecia was not improved. However, after the first-line treatment of CsA, the patient achieved a benefit of partial response, and his alopecia improved significantly after discontinuation of CsA. Moreover, we systematically analyzed the FDA Adverse Event Reporting System (FAERS) database for reports of alopecia with calmodulin inhibitors, including CsA and tacrolimus.

### Case description

**FIGURE 1:**
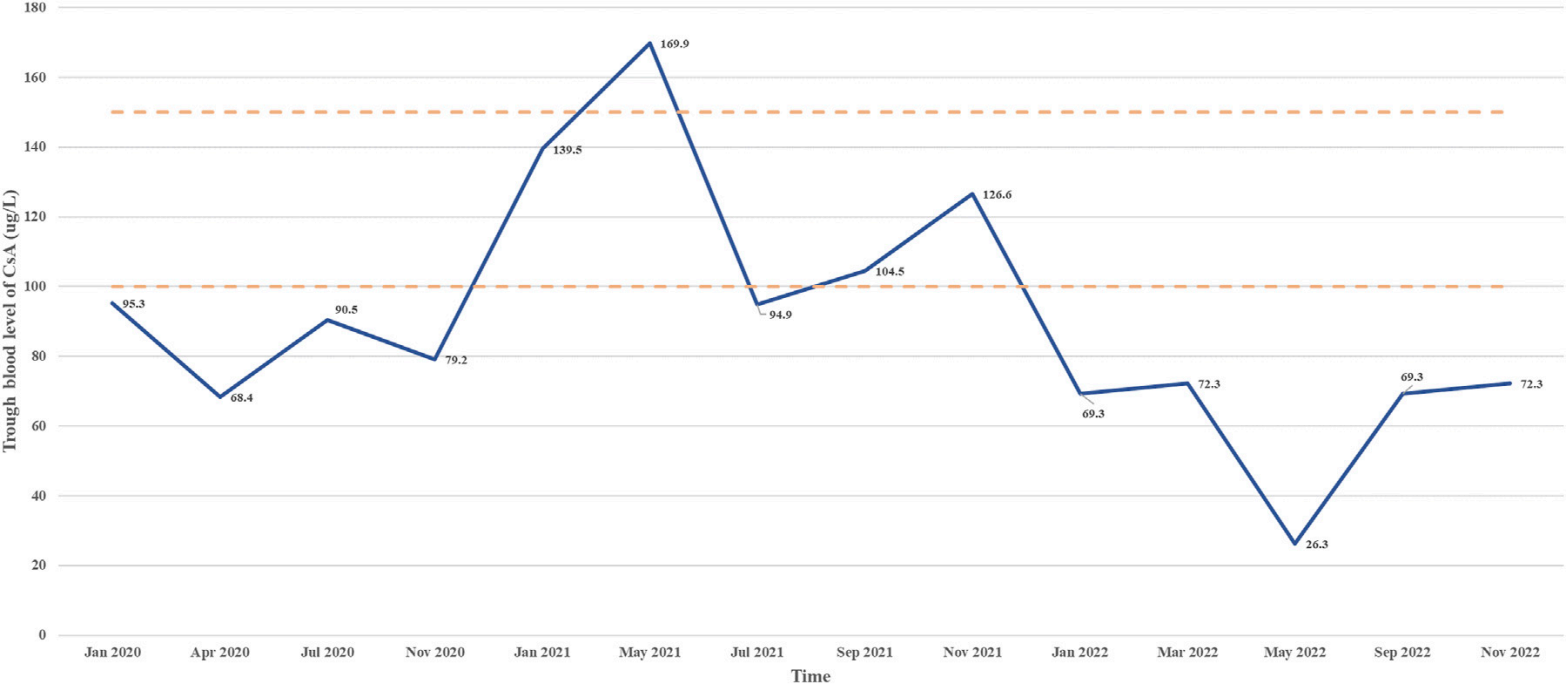
Trough blood level of CsA from January 2020 to May 2022.

**FIGURE 2:**
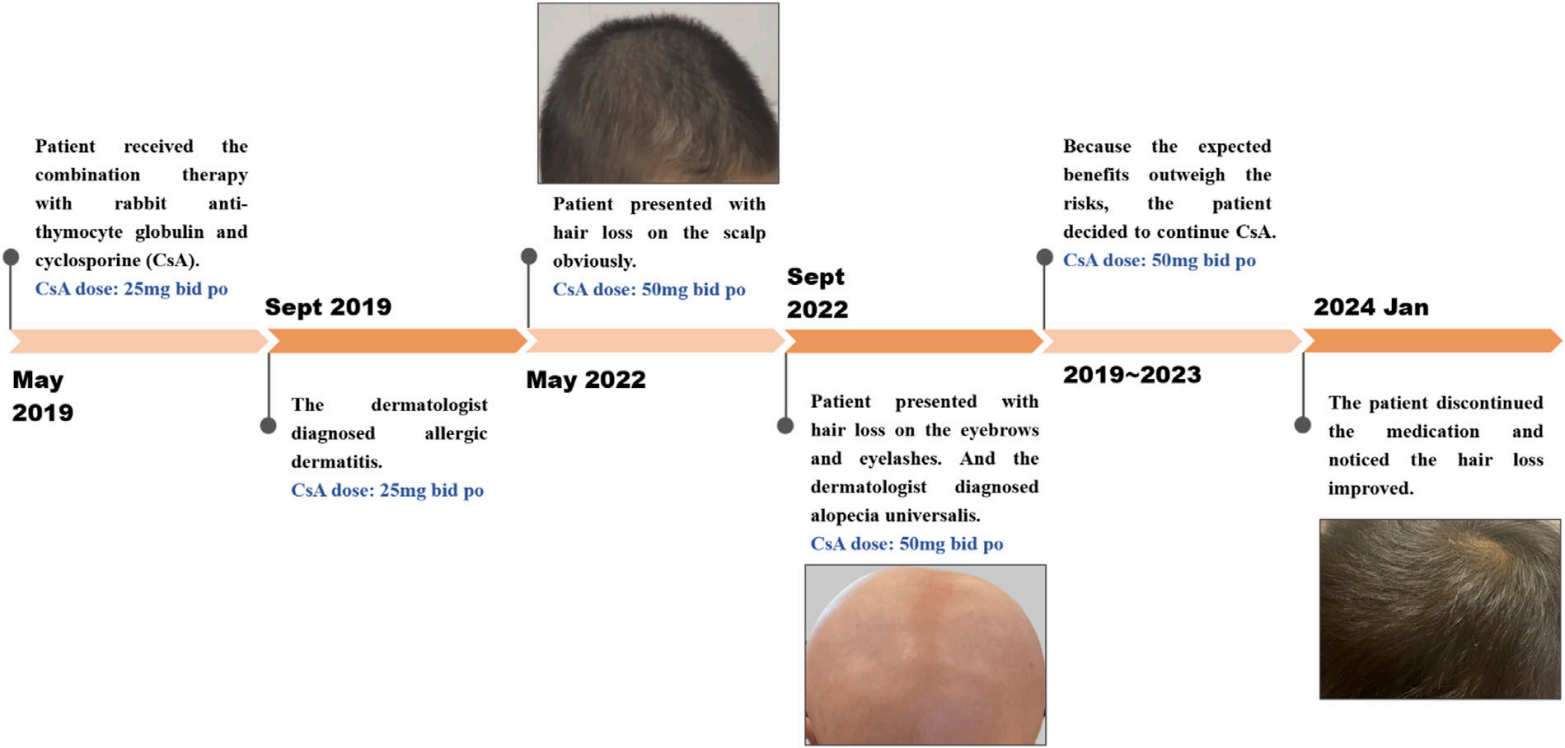
Timeline of alopecia report after exposure to CsA: The case is a 29-month-old boy who developed medication-induced hair loss during cyclosporine therapy for aplastic anemia.

A 29-month-old boy was diagnosed with severe aplastic anemia in May 2019. Treatments were initiated soon after diagnosis, including rabbit anti-thymocyte globulin and CsA. CsA was given orally at 5–10 mg per kilogram of body weight per day in two divided doses and then adjusted to a concentration within the appropriate range or an excellent clinical response (Figure 1; Table 1). Four months after the initiation of CsA, the patient was diagnosed with allergic dermatitis. In May 2022, he presented with hair loss on the scalp, which spread to the eyebrows and eyelashes (Figure 2). The patient did not report any pain or pruritus of the scalp, and he had no personal or family history of hair loss. Aside from CsA, his medical history was insignificant except for aplastic anemia under treatment with compound Zaofan pill, stanozolol tablets, folic acid tablets and mecobalamin tablets. Compound Zaofan Pill is a traditional Chinese herbal formula that can protect the kidney and marrow effectively in patients with aplastic anemia, leukopenia, thrombocytopenia, and so on (Yang et al., 2015). In the dermatological examination, the diagnosis of alopecia universalis with no associated inflammation was confirmed. Unfortunately, a cutaneous biopsy was not performed. Topical minoxidil liniment was administered to control hair loss. However, the child did not exhibit improvement following interventions. Although hair loss reduced the patients’ quality of life, the family decided to continue CsA because the expected benefits outweighed the risks. For follow-up, although the hair loss persisted throughout treatment, the disease showed an excellent response to CsA therapy. The patient discontinued the medication and noticed that the hair loss had improved.

**TABLE 1:** Patient pathological feature and clinical response.

Time	HGB (g/L)	PLT (10^9^/L)	NEUT (10^9^/L)	RET (10^12^/L)	Clinical response
Jan 2020	70	18	0.86	0.024	NR
Apr 2020	88	24	1.73	0.065	PR
Jul 2020	98	35	1.66	0.049	PR
Nov 2020	91	38	1.42	0.069	PR
Jan 2021	97	42	1.13	0.068	PR
May 2021	89	54	1.99	0.055	PR
Jul 2021	88	56	2.8	0.0597	PR
Sep 2021	84	73	1.6	0.0624	PR
Nov 2021	86	81	2.56	0.0496	PR
Jan 2022	91	85	1.78	0.046	PR
Mar 2022	94	74	1.77	0.055	PR
May 2022	88	68	2.13	0.05	PR
Sep 2022	99	97	2.25	0.0304	PR
Nov 2022	103	85	2.98	0.031	PR

Abbreviations: HGB, hemoglobin; PLT, platelet; NEUT, neutrophils; RET, reticulocyte; NR, none response; PR, partial response.

In this case, we could not exclude hair loss due to the natural course of the disorder or the effect of concomitant medications/drug interactions. However, there was a temporal relationship between CsA initiation and alopecia onset, supporting the potential role of CsA in promoting hair loss.

## Discussion

**FIGURE 3:**
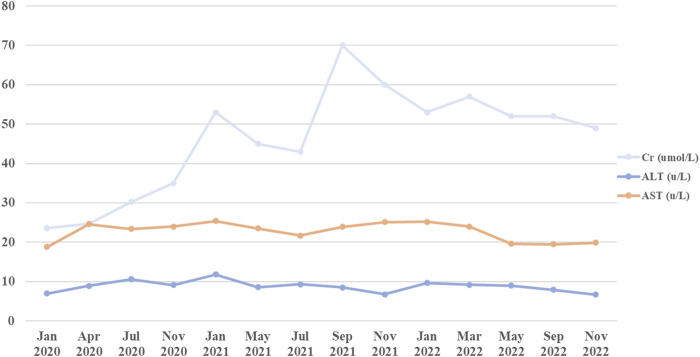
The liver & kidney indicators during CsA treatment.

CsA, a widely used immunosuppressant, has long been used to treat systemic lupus erythematosus (Subspecialty Group of Immunology et al., 2021), nephrotic syndrome (Trautmann et al., 2020) and aplastic anemia (Samarasinghe et al., 2018) in children. However, potential toxicity may limit its usage. Reversible hypertrichosis is a well-known cutaneous side effect of CsA, and has been recognized as a potent hair growth stimulator in both humans and rodents. Previous studies have shown that CsA may induce anagen in telogen hair follicles and inhibit hair follicle regression by blocking NFATc1, NFATc2, respectively (Paus et al., 1989; Paus et al., 1994; Horsley et al., 2008; Fujimura et al., 2011). Recently, CsA has been thought to target the hair follicles and promote hair growth by suppressing the secreted frizzled-related protein 1 (Hawkshaw et al., 2018). However, CsA has been implicated in alopecia with an incidence of 0.78%, recently (eHealthMe, 2024). Herein, we report a case of a 29-month-old boy who developed hair loss following administration of CsA. The patient had not been exposed to another culprit drug prior to alopecia. Although the aplastic anemia improved, the alopecia progressed rapidly to alopecia universalis. The patient presented in May 2022 with a hemoglobin concentration of 8.8 g per deciliter, a platelet count of 68,000 per microliter, and a neutrophil level of 2,130 per microliter. Alopecia areata is a multifactorial autoimmune disease that involves several factors, such as genetics, environmental factors, comorbidities, endocrine abnormalities, and drug consumption in combination or alone (Simakou et al., 2019; Dainichi et al., 2023). Drug-induced hair loss is a common cause of alopecia that can progress to total loss of scalp hair and body hair (alopecia universalis) (Simakou et al., 2019; Dainichi et al., 2023) and presents with a wide range of clinical phenotypes, including anagen effluvium, telogen effluvium, alopecia areata and scarring alopecia (Tosi et al., 1994; Alhanshali et al., 2023). CsA has been associated with induction of anagen effluvium (Saleh et al., 2023). In anagen effluvium, hair loss typically occurs within days to weeks of drug administration, affecting all the hair-bearing areas on the body, including the eyebrows, eyelashes and body hair (Tosti and Pazzaglia, 2007). However, in our case, the adverse reaction occurred approximately 3 years after starting CsA, and the delay from initial drug intake to index day was similar with the review of the literature (Todberg et al., 2020; Brunasso et al., 2023; Bourkas and Sibbald, 2022). For example, a case report describes one patient who experienced rapid hair loss after 2 years of starting CsA (Todberg et al., 2020). Bourkas AN (Bourkas and Sibbald, 2022) reported that a patient used CsA 5 mg/kg/day for atopic dermatitis with a noticeable improvement. However, new hair loss on the scalp and eyebrows after 7 months of CsA. CsA has been reported to have a high inter- and intraindividual pharmacokinetic disposition. In children, body weight, ontogeny of enzymes and total bilirubin level would affect the disposition of the drug in the body (Gao et al., 2022). Thus, CsA blood trough concentration was monitored without hepatotoxicity and nephrotoxicity (Figure 3). Most adverse drug reactions of CsA correlate with the single dose level, cumulative dose, and duration of treatment, and can be alleviated after dose reduction (Roy and Cyert, 2020; Kim et al., 2022). The duration between CsA used and the onset of alopecia suggests that a higher cumulative dose of CsA may increase the risk of hair loss.

Although numerous retrospective observational studies and case-control studies report alopecia as an adverse effect of carbamazepine, adalimumab, erenumab and so on (Rathore et al., 2021; Ruiz et al., 2023; Mounessa et al., 2023; Alhanshali et al., 2023), the mechanisms involved in drug-associated alopecia remain unclear. CsA’s action and potential regulatory mechanisms in alopecia have not been revealed. Alopecia onset and progression are strongly influenced by the inflammation and apoptosis around hair follicles (Simakou et al., 2019; Dainichi et al., 2023). On the one hand, CsA can activate or inhibit the immune system in dose-dependently (Flores et al., 2019). It has been shown that high-dose CsA inhibits T-cell activation, and low-dose CsA can induce pro-inflammatory cytokines, such as IFN-γ. IFN-γ impaired the immune privilege of hair follicles and interferes with the hair growth cycle by regulating the MHC molecule and JAK/STAT signaling, inducing alopecia areata finally (Paus et al., 2018; Simakou et al., 2019; Flores et al., 2019; Paggioli and Moss et al., 2022; Dainichi et al., 2023). On the other hand, CsA inhibits T-cell activation by inhibiting calcineurin phosphatase activity, thus suppressing transcription factor NF-κB. NF-κB, a vital hair growth regulator, is required for anagen maintenance in human hair follicles *in vitro* (Kloepper et al., 2014). The inhibition of NF-κB promoted apoptosis-driven catagen development in human anagen hair follicle. Therefore, we hypothesized that the hair-loss-stimulatory effects of CsA may be inflammation-induced by NF-κB. Moreover, the more precise mechanism of CsA in alopecia needs to be investigated.

Because no more than five cases of alopecia reaction secondary to CsA have been reported in the literature, we used the FAERS database to explore the potential role of CsA in hair loss (Table 2). According to FAERS, a total of 75,408 adverse reaction reports for CsA. We found an association between CsA and alopecia within these adverse reactions. In monotherapy, cyclosporine-induced alopecia was observed in 118 cases, and the adverse reaction in the drug combination group obviously increased. The prevalence of alopecia for CsA increased from 1.8% in 2004–2008 to 65.5% in 2019–2023, emerging as an adverse event to CsA.

**TABLE 2:** Report of alopecia with calcineurin inhibitor according to FAERS.

	Cyclosporine N (%)	Tacrolimus N (%)
Total Reports	731	745
Age, years		
Mean ± SD	45.5 ± 11.42	43.3 ± 13.29
≤18	20 (2.7)	49 (6.6)
18<n<45	123 (16.8)	113 (15.2)
45 ≤ n ≤ 60	397 (54.3)	393 (52.8)
>60	43 (5.9)	54 (7.2)
Not Specified	148 (20.2)	136 (18.2)
Sex		
Male	66 (9.0)	91 (12.2)
Female	613 (83.9)	617 (82.8)
Not Specified	52 (7.1)	37 (5.0)
Concomitant Medications (top 5)		
None	118 (16.1)	197 (26.4)
Tacrolimus	325 (44.5)	—
Cyclosporine	—	314 (42.1)
Infliximab	356 (48.7)	270 (36.2)
Mycophenolate Mofetil	313 (42.8)	364 (48.9)
Etanercept	380 (51.9)	302 (40.5)
Sirolimus	310 (42.4)	316 (42.4)
Daclizumab	272 (37.2)	278 (37.3)
Received Date		
2019–2023	479 (65.5)	458 (61.5)
2014–2018	180 (24.6)	219 (29.4)
2009–2013	59 (8.1)	50 (6.7)
2004–2008	13 (1.8)	18 (2.4)
Severity		
Non-serious	91 (12.4)	144 (19.3)
Serious	640 (87.6)	601 (80.7)
Reporting Source		
Healthcare professional	146 (20.0)	198 (26.6)
Consumer	575 (78.7)	528 (70.9)
Not Specified	10 (1.3)	19 (2.5)

Our case report has limitations, particularly in dermatological diagnosis, due to the absence of a cutaneous biopsy. Thus, we could not confirm the drug-induced etiology definitively.

## Conclusion

To summarize, we present a rather unusual case of hair loss that developed during CsA administration, while other literature reported a paradoxical reaction. Given the widespread use of CsA in pediatrics, we hope to improve recognition and management of this adverse drug reaction by sharing this case.

## Methods

### Data sources

We extracted calmodulin inhibitor reports from the FDA Adverse Event Reporting System (FAERS) database. FAERS is a database containing adverse event reports, medication error reports, and product quality complaints from adverse events submitted to the FDA. Healthcare professionals, consumers, and pharmaceutical companies submitted the adverse event reports. In this study, data on calmodulin inhibitors and alopecia were extracted from the databases between January 2004 and January 2023. We eliminated the duplicated reports with all the same values in the fields “sex”,“age”, “case ID”, “event date”, “adverse reaction” and “Reporter Type”. In addition to analyzing drug adverse reactions, demographic factors such as gender and age, reporting source and concomitant medications were also taken into consideration.

We have searched PubMed using the entry “(alopecia OR hair loss) AND (ciclosporin OR cyclosporin)”. The article describing any form of alopecia following exposure to cyclosporin can be included. Exclusion criteria were as follows: 1) no full text electronically available; 2) study type: animal studies, *in vitro* experiments, systematic reviews or meta-analyses; 3) articles not published in the English language.

## Data Availability

The original contributions presented in the study are included in the article/supplementary material, further inquiries can be directed to the corresponding author.
